# Development of a cardiac-centered frailty ontology

**DOI:** 10.1186/s13326-019-0195-3

**Published:** 2019-01-18

**Authors:** Kristina Doing-Harris, Bruce E. Bray, Anne Thackeray, Rashmee U. Shah, Yijun Shao, Yan Cheng, Qing Zeng-Treitler, Jennifer H. Garvin, Charlene Weir

**Affiliations:** 1 Nuance Communications, Burlington, MA USA; 2 Division of Cardiovascular Medicine, University of Utah, Salt Lake City, UT USA; 3 Department of Biomedical Informatics, University of Utah, Salt Lake City, UT USA; 4 Physical Therapy and Athletic Training Department, University of Utah, Salt Lake City, UT USA; 5 Medical Informatics Center, George Washington University, Washington DC, USA; 6 VA Healthcare System, Salt Lake City, UT USA

**Keywords:** Ontology, Frailty, Surgery, Cardiology, SNOMED-CT

## Abstract

**Background:**

A Cardiac-centered Frailty Ontology can be an important foundation for using NLP to assess patient frailty. Frailty is an important consideration when making patient treatment decisions, particularly in older adults, those with a cardiac diagnosis, or when major surgery is a consideration. Clinicians often report patient’s frailty in progress notes and other documentation. Frailty is recorded in many different ways in patient records and many different validated frailty-measuring instruments are available, with little consistency across instruments. We specifically explored concepts relevant to decisions regarding cardiac interventions. We based our work on text found in a large corpus of clinical notes from the Department of Veterans Affairs (VA) national Electronic Health Record (EHR) database.

**Results:**

The full ontology has 156 concepts, with 246 terms. It includes 86 concepts we expect to find in clinical documents, with 12 qualifier values. The remaining 58 concepts represent hierarchical groups (e.g., *physical function findings*). Our top-level class is *clinical finding*, which has children *clinical history finding*, *instrument finding*, and *physical examination finding*, reflecting the OGMS definition of clinical finding. *Instrument finding* is any score found for the existing frailty instruments. Within our ontology, we used SNOMED-CT concepts where possible. Some of the 86 concepts we expect to find in clinical documents are associated with the properties like *ability interpretation*. The concept *ability to walk* can either be *able, assisted* or *unable*. Each concept-property level pairing gets a different frailty score. Each scored concept received three scores: a frailty score, a relevance to cardiac decisions score, and a likelihood of resolving after the recommended intervention score. The ontology includes the relationship between scores from ten frailty instruments and frailty as assessed using ontology concepts. It also included rules for mapping ontology elements to instrument items for three common frailty assessment instruments. Ontology elements are used in two clinical NLP systems.

**Conclusions:**

We developed and validated a Cardiac-centered Frailty Ontology, which is a machine-interoperable description of frailty that reflects all the areas that clinicians consider when deciding which cardiac intervention will best serve the patient as well as frailty indications generally relevant to medical decisions. The ontology owl file is available on Bioportal at http://bioportal.bioontology.org/ontologies/CCFO.

**Electronic supplementary material:**

The online version of this article (10.1186/s13326-019-0195-3) contains supplementary material, which is available to authorized users.

## Background

### Frailty and cardiac decision making

Frailty is an important patient attribute for treatment decisions in general [1–4] because assessing frailty severity predicts response to treatment and patient outcomes across many conditions [2, 5–10]. In the modern era of interventional cardiac care, patient frailty is increasingly important to decisions regarding major cardiac surgery and interventional procedures [1, 9, 11]. With the growing numbers of elderly and diabetic patients [6, 12], these decisions are common [13]. Older, frail patients with aortic valve stenosis can now be referred for coronary artery bypass graft surgery (CABG), a transcatheter valve replacement (TAVR), or medical management [14–16]. While TAVR is minimally invasive with shorter length of stay, frail patients may not necessarily benefit due to non-cardiac illnesses that limit quality of life or increase risk of procedural complications [11, 17], including increased length of stay, infection rates, and re-hospitalization. In 2015, the National Institute on Aging cited frailty assessment as a key priority in the perioperative approach to cardiac surgery [13].

Assessing frailty is done by intuitive estimates or appraisals, counting comorbid conditions, and the use of formal assessment instruments [2, 18, 19]. Frailty can include physical disability, deficits in mood, sensorium, and cognition, along with patient experience of pain or incontinence [3, 6].

The purpose of this paper is to describe the development of an ontology of frailty, paying special attention to how it relates to cardiac care decisions. Our ontology is designed to access the aspects of frailty that distinguish it from a simple count of comorbid conditions. We describe a necessary and sufficient view of patient frailty indicators apart from comorbid conditions. This ontology has been designed to allow computerized extraction of frailty information from the narrative documents patient records. Because frailty is a topic that is interpreted in many ways and measured with several instruments [5, 18–20], we built our ontology using as many term identification techniques as possible, gathering terms using existent instruments, physician interviews, and automated chart reviews. We aimed to allow cross walking between the measurement instruments. The hierarchical structure was adapted from SNOMED-CT [21] but expanded and informed by the nuances in clinical decision-making. We tested a draft version of our ontology by creating instrument-scoring rules, by using it to improve automated detection of frailty indicators in a Natural Language Processing (NLP) system, and by using it as an input feature to a system trained to predict patient mortality after a major cardiovascular procedure (MCVP) [22].

### Frailty and NLP

Given that frailty information is so important, extracting it from clinical records is vital for patient care and research. The three methods of extracting information from clinical records are structured data, human chart review, or automated NLP systems. There are 3 reasons why an NLP approach is likely to be the most successful: 1) physicians do not consistently use frailty instruments, 2) there is no key, which reconciles scores across instruments, 3) they do not all use the same definition of frailty.

Clinicians collect frailty information, but not in a systematic fashion nor by consistently using frailty instruments [20]. They document narrative descriptions of frailty information that they find relevant to the specific clinical situation. It is possible that since clinicians believe they can rapidly use their clinical judgment to assess a patient’s frailty when they see them [18], they do not feel the need to systematically use specific frailty instruments. Their narrative notations are considered sufficient. However, large-scale retrospective studies of patient outcomes require chart review, and if frailty is largely documented in narrative, then structured text cannot be used and the effort of wading through text in a chart causes a time bottleneck for human reviewers.

The inconsistent use of frailty instruments would not matter for chart review based on structured information if there were a method for reconciling scores from different instruments. The method would create equivalences between the instruments. These equivalences would take as many factors into account as possible. Creating score equivalence metrics would be a task that humans would find challenging.

If clinicians all used a similar definition of frailty, humans chart review or NLP systems without ontology components would be able to locate their descriptions easily, but they do not. Clinicians’ ideas about which patients are frail are influenced by both the culture within their organizational department, the decision at hand, and the wider society. For example, departmental culture may involve specific frailty tests (e.g., 6-min walk distance) and social culture may mean that frailty indicators have different thresholds (e.g., low body mass index (BMI) in Japan vs. the US [23].) Frailty indicators are also specific to each patient. A patient’s level of mobility is highly dependent on prior exercise activities, desire for exercise, and the patient’s personal preferences. The number of frailty instruments that have been developed evidences the variability in the conception of frailty, and therefore the complexity of the relationship between frailty and decision-making. Buta, et al. [20] identified 67 frailty instruments of which nine were cited more than 200 times.

Since current charting practices make human chart review or using structured data untenable, one could force a structured data solution by picking a single instrument and screen all patients. Picking a single instrument and screening everyone is hampered by the low specificity of current instruments [4, 20] and by lack of instrument adoption. In addition, frailty assessment varies substantially over time. Assessing all individuals over time would be necessary to understand the trajectory and implications of frailty [4], which would mean that the structured data solution would only become helpful after a significant time-interval. Systematically conducting frailty assessments at all encounters would fail to highlight decision-specific frailty issues, it would add a substantial burden to the clinician, and cost to the healthcare system.

### Ontology building

Ontologies may be used in NLP projects to bridge structured data fields. Some structured data fields from clinical records across institutions or even within the same institution use different words to denote the same information (e.g., “patient name” vs. “lastname, firstname”) [24–26]. Ontologies are also used for named entity recognition and decision modeling [27–29]. For example, named entity recognition can locate all mentions of disorders that patients may have as well as relevant patient demographics. Decision modeling uses either the named entities found or other inputs to access ontological elements, which contribute to creating rules or other models of decisions. Our ontology of patient frailty is designed to fulfill both purposes.

We employed the standard methodology for building ontologies including reconciling clinical text, medical literature, and existing ontologies [26, 30–32]. We chose to develop our ontology by adhering as closely as possible to realist principles. Realist principles lead to stable ontologies [33], which can be reasoned with while avoiding illogical inferences [34].

We conceptualized clinical records as textual recordings of the author’s *ideas* about the patient. An existing ontological concept that corresponds to the author’s ideas about the patient is *clinical finding* from the Ontology of General Medical Science (OGMS), which is defined as “A representation that is either the output of a clinical history taking or a physical examination or an image finding, or some combination thereof.” [35] In contrast, the definition of a *clinical finding* used in SNOMED-CT is “observations, judgments or assessments about patients.” The definition specifies that it is designed to convey “…the actual state of the body” and is inclusive of concepts with a semantic tag *disorder* (http://browser.ihtsdotools.org/). By referring directly to the patient’s body and not the clinician’s findings, one is ignoring consideration of human error, cognitive biases, and other aspects that may influence patient-clinician and clinician-EHR interactions [36, 37]. However, SNOMED-CT’s definition of *clinical finding* also includes concepts with the semantic tag *finding*, which “…are not separate from the observing of them,” which brings them closer to the OGMS definition. We restricted ourselves to *findings*.

### Integration with prior work

Two prior studies have successfully mined frailty information from rehabilitation and nursing home notes. One generated International Classification of Functioning, Disability and Health (ICF) codes and the other extracted Barthel index scores [38, 39]. Their work indicates that it is possible to locate and extract frailty-relevant terms. We expanded their work by increasing the number of frailty-related terms identified.

The UMLS Metathesaurus [40] contains a complex structure of frailty-related concepts. It is evident that SNOMED CT contributes concepts from many, if not all, of the available frailty instruments. However, the UMLS Metathesaurus is not realist due to long-standing requirements of backward compatibility [33, 41, 42]. We wanted our ontology to be interoperable with as many other ontologies as possible. We did not want to create something that entirely ignored the UMLS Metathesaurus. Recent papers have discussed realist approaches, specifically with respect to SNOMED-CT [33, 41, 43, 44]. Compatibility with SNOMED-CT can be used as a bridge to the UMLS Metathesaurus. Therefore, we incorporated SNOMED-CT concepts into the Cardiac-centered Frailty Ontology as often as possible, but did not limit ourselves to SNOMED-CT.

### Objective

In this study we created a machine-interoperable description of frailty that reflects all the areas that clinicians consider when deciding which cardiac intervention will best serve the patient as well as general indications of frailty found in patient records.

## Results

In this section we describe each of the four phases of ontology development (Identify other ontologies and official clinical tools, group terms into high-level classes, define attributes of classes, analyze and validate), which led to the final ontological structure.

### Phase 1 – Collect terms by identifying other ontologies and official clinical tools

The research team met regularly to iteratively identify terms from a variety of sources. We reviewed 14 frailty instruments described in the methods section (below). The terms from these instruments were mapped onto the UMLS meta-thesaurus. If there was a SNOMED-CT term we used it.

In addition, we interviewed 12 clinicians (cardiologists, geriatricians, and cardiac surgeons) where we provided 7 hypothetical patient vignettes. Clinicians were asked to discuss patient frailty in relation to a decision between CABG, TAVR, and medical management. Each hypothetical patient had a different mix of frailty indicators. The full study will be described in a separate paper. The terms found in the interviews included *muscle weakness, oxygen need, gait velocity, 6-min walk distance, volunteers, lower extremity strength, robust, functional status, functionality, deconditioned, acute* vs. *chronic findings* and *BMI*. We identified terms at the level of concept granularity relevant to cardiac decision-making. For example, terms relating to housework (e.g., “dusting,” “washing dishes,” and “vacuuming”) were grouped into a single concept *ability to perform domestic activities* because whether a patient is dusting or vacuuming is not relevant to their cardiac health.

In order to filter the terms into unique groups to aid the next step of creating hierarchies, the total set of terms underwent an initial sorting by the research team to identify explicit synonyms and concepts. The terms were found to correspond to the general categories of toileting, mental health, social functioning, working, exercise, walking, eating, general health, bathing, dressing & grooming, transfers, and modifiers (i.e., body locations and qualifiers). Using these groupings, we had an initial set of 108 unique concepts.

For those 108 concepts, we identified 198 unique concept-related terms. Terms within concepts were further expanded by SNOMED-CT synonyms and augmented by the team’s previous experience with clinical documents. After the term expansion, nearly all of the concepts had either 1 or 2 terms, while one concept had 12 terms. The concept with the most terms was *Lack of energy finding*, with “lack of energy,” “tired,” “fatigue,” “lack energy,” “tiredness,” “sleepiness,” “drowsiness,” “exhaustion,” “exhaust,” “wear out,” “drain,” and “weary.”

Our clinician interviews made it clear that clinicians rely heavily on the Society of Thoracic Surgeons (STS) score in assessing patient’s likelihood of surviving surgery. We examined the STS calculation and found it was more sensitive to comorbid conditions than indications of frailty. The clinicians also indicated that comorbid conditions are often included in assessments of patient frailty. Since we were interested in creating an ontology of frailty apart from comorbid conditions, we only included the concept *comorbid conditions count*, not specific conditions the patient might have.

A second term expansion was done using automated chart review. Terms found included “stagger” as indicative of the concept *impairment of balance finding*, “prosthetics,” “shoes,” “gripping strength,” “fatigue,” “weakness,” “SOB,” “short of breath,” “dyspnea,” “muscle strength,” “motor strength,” “decreased strength,” “assist,” “paralyzed,” “handicap,” “unassisted,” “dresses,” “bathes,” and “stand.” Some terms were removed because they commonly occurred with a meaning alternate to the one we were after. These terms included “dressing,” “supine,” “working,” “eating,” “strength,” “incontinence.”

After all terms had been gathered we had 246 terms associated with the 108 concepts.

### Phase 2 – Group terms into high-level concepts

The identified frailty concepts were arranged in hierarchical relationship by mapping them to SNOMED-CT equivalents using SNOMED-CT concepts within the category *clinical finding*, with semantic type *finding*. The goal for this process was to restrict the SNOMED-CT mappings to as small a selection as possible, while maintaining correspondence, which means our ontology is somewhat compatible with SNOMED-CT.

**Fig. 1:**
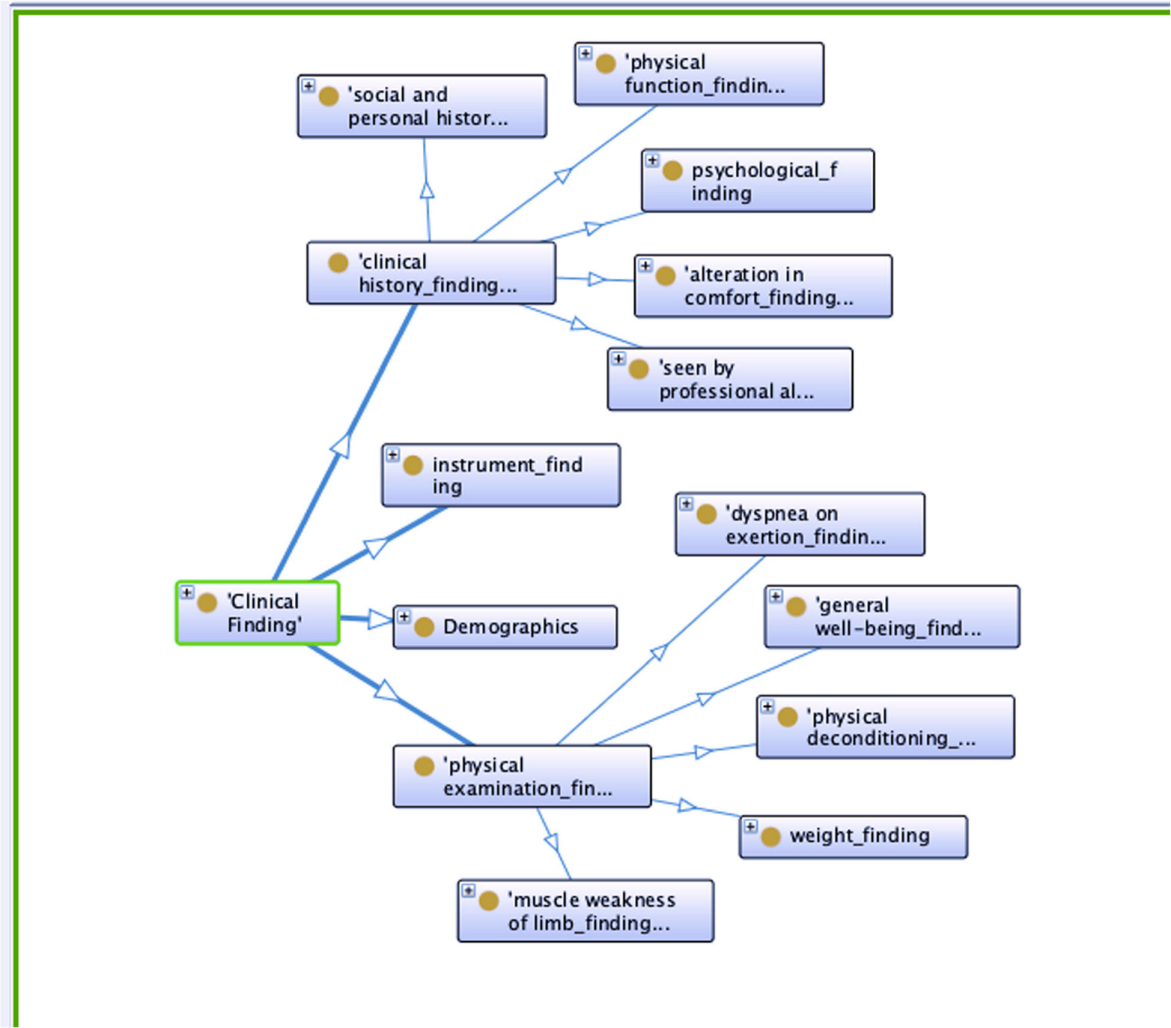
Top three layers of concepts in the Cardiac-centered Frailty Ontology

Figure 1 shows the top concepts in our Cardiac-centered Frailty Ontology. In order to create an ontology that is interoperable with SNOMED-CT, it is important that where concepts in the two ontologies share a name and an id number they are used to represent precisely the same portion of reality. Therefore, if we did not match the exact use described by SNOMED-CT, we explain why and do not use the SCTID.

As discussed in the introduction, we did not use *clinical finding* in the same way as SNOMED-CT, we restricted ourselves to the subset with semantic tag *finding*. Therefore, we did not use the SNOMED-CT ID number for the concept *clinical finding*. Our concept *Clinical finding* (CCFOID:1) has children *clinical history finding* (CCFOID:11), *instrument finding* (CCFOID:12), and *physical examination finding* (CCFOID:13), reflecting the OGMS definition of clinical finding. *Instrument finding* is any score found for the existing frailty instruments already mentioned. We included classes not mapped to SNOMED-CT for *demographics* (CCFOID:14) and *qualifier values* for the properties of our concepts in our top level.

In the Cardiac-centered Frailty Ontology we included *demographics* in *clinical findings* because we are referring to demographic information collected by the clinician at a clinical visit, not to the demographic information that inheres in the patient and may change between visits.

#### Clinical history finding

The obvious choice for findings arising from the patient’s clinical history taking is *clinical history and observation finding (finding)* (SCTID: 250171008). It turns out that all of the children listed in the SNOMED-CT browser (http://browser.ihtsdotools.org) have semantic type *finding*, which is what we were after. Like *clinical finding*, we do not want to include all the *children of clinical history and observation finding,* which means we are not referring to the same portion of reality. Synonymous terms and the SNOMED-CT term identification number (SCTID) are not necessary because we are not looking for the concept to be represented in clinical documents. For these reasons, we shorten the name to *clinical history finding* and exclude the SCTID.

The same problem arises when we try to find a SNOMED-CT equivalent of *physical function finding* (CCFOID:112). The topic modeling, our interviews with clinicians, and existing instruments all indicate that the patient’s physical abilities are a necessary category. The closest SNOMED-CT equivalent is *functional finding (finding)* (SCTID: 118228005), which has among its children concepts we need, for example *finding of activity of daily living (finding)* (SCTID: 118233009, CCFOID:1124). However, it includes findings unrelated to physical abilities like *does comply with treatment* (SCTID: 386673006). Therefore, we do not use *functional finding* and leave *physical function finding*, with no SCTID.

SNOMED-CT is so exhaustive that there can be hierarchical structure that is beyond our needs. *Social and personal history finding* (SCTID: 365448001, CCFOID:115) has two intervening problem parents *finding by method* and *history finding*, which lead to *clinical finding* and not *clinical history and observation finding*. There is no indication in the documentation about how *history finding* differs from *clinical history and observation finding*. The children of *Social and personal history finding* and *psychological finding* (SCTID: 116367006, CCFOID:113) that we are interested in also have intervening concepts. It may be better practice to just use the SCTIDs for the lowest levels. Those will be the only ones used in the NLP.

*Alteration in comfort finding* (SCTID: 130979001, CCFOID:111) has the intervening parent problem *sensory nervous system finding (finding)* (SCTID: 106147001) and *neurological finding (finding)* (SCTID: 102957003), which go to *clinical finding (finding)*. The *courses qualifier value* (CCFOID:43) concept we include corresponds to the SNOMED *clinical finding* attribute *clinical course*. We also include a concept for *seen by a professional allied to medicine finding* because the snippet annotations indicated that being seen by physical therapy, occupational therapy or other allied professions indicated patient frailty.

#### Physical examination finding

We included in *Physical Examination Finding* (CCFOID:13) concepts that fall in the SNOMED-CT hierarchy under *general findings of observation of patient (finding)* (SCTID: 118222006). In SNOMED-CT general finding of observation of the patient is a child of *clinical history and observation finding*. Since we did not include *observation* in our concepts, we included this separate concept for physically observing the patient. We took a very restricted subset of the children of *general findings of observation of patient (finding)*, hence the name change and absence of SCTID. The children we included are *physical deconditioning finding* (SCTID: 31031000119102, CCFOID:134), *dyspnea on exertion finding* (SCTID: 60845006, CCFOID:131), *muscle weakness of limb finding* (SCTID: 713514005, CCFOID:133), *weight finding* (SCTID: 107647005, CCFOID:135), and *general well-being finding* (SCTID*:* 365275006, CCFOID:132).

*Physical deconditioning* has no children. We included all of the children of muscle weakness of limb because they separate upper and lower limbs, which our interviews indicated is an important distinction. *Weight finding* has many irrelevant children including *finding of color zone for Broselow Luten pediatric weight estimation (finding)*. We did not include these children.

For *dyspnea on exertion finding,* and *General well-being finding,* we kept the SCTID because we could map all of the children, although this is not currently part of the ontology. For the children not currently explicitly listed, we would need to determine whether they were indicative of high or low frailty. We added the concept *comorbid condition count finding* (CCFOID:131), which we discussed earlier, as a child of *general well-being finding*.

The full ontology has 156 concepts, with 246 terms. The ontology owl file is available on Bioportal at http://bioportal.bioontology.org/ontologies/CCFO. We consider CCFO a “view” into SNOMED-CT. We define “view” in accordance with the Ontology Views Project being done by the Structural Informatics Group at Washington University (http://sig.biostr.washington.edu/projects/ontviews/). In this definition a view is a new ontology that includes some portion of the viewed ontology. CCFO contains portions of SNOMED-CT. It is therefore a view of SNOMED-CT. As a view, it falls under SNOMED-CT’s existing licensure (https://www.snomed.org/snomed-ct/get-snomed).

Table 1 shows the number of concepts by their number of terms. The table lists the number of terms associated with each of the 86 concepts we expect to find in clinical documents. The remaining 58 concepts, not in the table, represent hierarchical groups (e.g., *physical function findings*) and 12 *qualifier values*. Two concepts from *demographics* (CCFOID:14) have no terms (*patient age finding*, CCFOID:141 and *indeterminate sex finding*, CCFOID:1422). *Lack of energy finding* (CCFOID:132222) still has the highest number of terms.

**Table 1:** Breakdown of the number of terms per concept in the Cardiac-centered Frailty Ontology. These counts are for the 86 concepts that we expect to find in clinical documents

# terms	1	2	3	4	5	6	7	8	> 8
# concepts	24	29	7	8	6	5	0	3	4

Table 2 lists some important concepts and their associated terms. The term list is included as Additional file 1. Since terms are not synonyms for the concepts in the ontology, they are not included in the ontology itself. Terms are text that we consider indicative of the author’s thoughts about the concept. Concepts themselves are portions of reality, not pieces of text.

**Table 2:** List of concepts central to assessing frailty and their associated terms. Terms are not synonymous with the concept or the concept name. They indicate author may have been thinking about the concept. Bolded terms were not found in the topic modeling paper. Underlined terms were added by the annotation task

Concept	Terms (not synonyms)
ability to run finding	**Difficulty running; able to run; unable to run; run**
ability to stand finding	Difficulty standing up; unable to stand up; able to stand up; stand up
able to mobilize finding	ambulate independently; steady gait; unsteady gait
bed-ridden finding	bed-ridden; supine; stretcher
Paralysis finding	paralysis; paralyzed
wheelchair bound finding	wheelchair; scooter; w/c; wheel chair
able to perform dressing activity finding	dresses; Able to dress; independent with dressing; Needs help with dressing; Dependent for dressing; unable to dress; Difficulty dressing; shoes; ties shoes;
able to perform personal grooming activity finding	Able to wash own hair; Unable to wash own hair; Difficulty washing own hair; clean appearance; personal grooming; neatly dressed; well-groomed; well-groomed without assistance; good personal hygiene

### Phase 3 – Define object properties for concepts

Concept properties were determined by rating scales used in the instruments. Activities have a frequency property that is found in the SNOMED-CT *frequency qualifier value* (SCTID: 272123002, CCFOID:44) restricted to *high frequency qualifier value* (SCTID: 27732004, CCFOID:441) and *mid-frequency* (SCTID: 255218000, CCFOID:442). Frequency values contrast with a value of *absent finding qualifier value* (SCTID: 272519000, CCFOID:42). Possible values are restricted based on the likelihood of finding specific text qualifiers. Abilities have an ability interpretation property that is found in *ability interpretation qualifier value* (SCTID: 371148001, CCFOID:41). These values are also restricted to *able qualifier value* (SCTID: 371150009, CCFOID:412), *able with difficulty qualifier value* (SCTID: 371157007, CCFOID:411) and *unable qualifier value* (SCTID: 371151008, CCFOID:413)*.* Finally, all clinical findings have a course property from *courses qualifier value* (SCTID: 288524001, CCFOID:43), including *chronic qualifier value* (SCTID: 90734009, CCFOID:431), *clinical course with short duration qualifier value* (SCTID: 424572001, CCFOID:432), and *sudden onset qualifier value* (SCTID: 385315009, CCFOID:433).

More properties of the concepts were determined by scores of relevance to cardiac decisions and their likelihood of resolving after the recommended intervention. Three investigators and three interview participants scored 81 of the 84 concepts that we expected to find in clinical documents. Three concepts were added after scoring was complete (*ability to drive a car finding* CCFOID:11241, *quadriceps weakness finding* CCFOID:1334, and *calf weakness finding* CCFOID:1331).

For the 81 concepts that were scored, ability concepts were qualified with the *able qualifier values* (able/independent, with difficulty/assisted, and unable/dependent) each concept-value pair was given a separate score. Activity and mental state concepts were qualified with *frequency qualifier values* (high frequency, mid-frequency, absent) and scored seperately. Rockwood categories as described in the Dalhousie University Clinical Frailty Score [45] were averaged across the eight raters. Ratings of low, medium, or high for relevance to frailty and fix-ability where set to the majority rating for the six raters, who had clinical experience.

Only three concepts were given low relevance to frailty ratings by all six raters *calm finding* (CCFOID:113331), *happy finding* (CCFOID:113332), and *nervous finding* (CCFOID:113333) concepts from the *mental state finding* (CCFOID:11333) concept. Fifty-two concepts were rated as highly relevant by all six raters, nine by at least three raters. Thirteen concepts had relevancy ratings of medium by all six raters, four by at least three raters. Table 3 shows the findings for nine concepts central to the assessment of frailty. *Ability to participate in leisure activities finding* (CCFOID:112412) is included in Table 3 to demonstrate a cardiac intervention-specific concept.

**Table 3:** Scores for nine concepts central to the assessment of frailty. Rockwood scores are on a scale of 1 - very fit to 9 – terminally ill. They are averaged across raters. “Will fix” refers to clinical findings that the cardiac intervention will alleviate. Relevance is how important the concept is to decisions about cardiac interventions. L – low, m – medium, h – high

Concept	Rockwood			Will Fix	Relevance
	Able	With Difficulty	Unable		
ability to run finding	1	3.33	4	M	H
ability to stand finding	2.67	5.11	7.44	TIED L+	H
able to mobilize finding	steady gait 3		unsteady gait 6	M	H
bed-ridden finding	Only level 8			L	H
Paralysis finding	Paraplegic 6		Quadriplegic 8	L	TIED M+
wheelchair bound finding	Only level 6			M	H
able to perform dressing activity finding	3	4	7	L	H
able to perform personal grooming activity finding	1	4	7	L	H
ability to participate in leisure activities finding	2	3	5.5	H	H

We determined which concepts are specific to cardiac intervention decisions by using the difference between ratings for *will fix* and *relevance* (in Table 3). *Will fix* refers to findings the cardiac intervention will alleviate; while *relevance* refers to findings that our reviewers indicated were relevant to frailty. We considered concepts that are highly relevant to frailty and are either highly likely to be alleviated by cardiac intervention or are associated with eventual recovery, to be especially important. For instance, bed-ridden is seen as generally relevant and not specifically relevant to cardiology. *Enjoys light exercise finding* (CCFOID:11231), *ability to participate in leisure activities finding* (CCFOID:112412), *dyspnea on exertion finding* (CCFOID:131), and *fit and well finding* (CCFOID:1323) are all rated as specifically relevant to cardiology as well as being generally relevant. Thirty-two concepts are rated as moderately specific to cardiology and 45 were given low cardiology-specific ratings.

In this section, we also looked at the mapping instrument scores found in the clinical document set to frailty scores from Rockwood categories as described in the Dalhousie University Clinical Frailty Score [45]. Table 4 lists the instruments and their scoring criteria.

**Table 4:** Instrument scores found in clinical document set and the scoring criteria, which allow the NLP system to use the scores to determine indication of frailty

Instrument Name	Scoring Criteria	
Activities of Daily Living (ADL) Screen	18 patient independent 6 patient very independent	
Functional Independence Measure (FIM)	7-complete independence; 6-modified independence; 5-Supervision or step-up; 4-Minimal Contact Assistance;	3-Moderate Assistance; 2-Maximal Assistance; 1-Total Assistance
Katz index ADL	Score of 6 = High, Patient is independent. Score of 0 = Low, patient is very dependent.	
Barthel index	ADL: 70–100 = Independent; Less than 70 = Needs significant physical/supervisory assistance.	
Instrumental activities of daily living (IADL)	2 = without assistance, 1 = with assistance, 0 = unable	
Instrumental activities of daily living (IADL) scale (Lawton) / IADL Screen	The total score may range from 0 to 8. A lower score indicates a higher level of dependence.	
Functional Activity Questionnaire (FAQ)	Score of 5 or more indicates significant impairment in instrumental activities of daily living.	
Morse fall scale / Annual Fall Scale / MRT	> = 45: high fall risk 25–44: moderate risk 0–24: low risk	
Tinetti assessment measures	Maximum possible balance score: 16 points. Maximum possible gait score: 12 points. Maximum total score: 28 points. -Scores below 19 indicate high risk for falls. Scores in the 19–24 range indicate some risk for falls.	
Braden scale	Pressure Ulcer Risk: total score < =9 very high risk total score 10–12 high risk	total score 13–14 moderate risk total score 15–18 mild risk total score 19–23 no risk

### Phase 4 - analyze and validate

We created implementation rules to map ontology elements to instrument questions for three common instruments. The rules for these three instruments (Barthel index, Katz ADLs, and SF-36) are listed in Table 5. Note that the mappings are not one-to-one. Some of the instrument questions were mapped to equivalent concepts. For example, both Barthel index and Katz ADLs uses the parent concepts *ability to perform personal care activities* (SCTID: 284774007) to include feeding self, dressing, grooming, toileting, and washing oneself, and *ability to transfer location* (SCTID: 714882001). By creating implementation rules, we were able to demonstrate that the Cardiac-centered Frailty Ontology covered the topics used in the instruments.

**Table 5:** Examples of Frailty Instruments implemented with the Cardiac-centered Frailty Ontology

Frailty Insrument: Barthell Index	
*Incontinence finding* (CCFOID:11271) of either kind = 0 *Continence finding* (CCFOID:11271) or *absent* qualified *incontinence finding* of both kinds = 2 *Able to perform personal care activities finding* (CCFOID:11244) for each of its children: unqualified or *able* = 2 *with difficulty* = 1 *unable* = 0 *Ability to transfer location finding* (CCFOID:1122)(any one) = 2 *Absent* (CCFOID:42) qualified = 0 *Able* (CCFOID:412) qualified *able to mobilize finding* (CCFOID:112121) or *no aid for walking finding* (CCFOID:112472) = 3 *Able with difficulty* (CCFOID:411) qualified *able to mobilize finding* or *walking aid use finding* (any kind) = 2 *wheelchair bound finding* (CCFOID: 1121223) = 1 *unable* (CCFOID:413) qualified *able to mobilize finding* or *bed-ridden finding* (CCFOID:1121221) = 0 *Able* qualified *able to walk upstairs finding* (CCFOID:1124713) or *able to walk downstairs finding* (CCFOID:1124711) = 2 *Unable* qualified *able to walk upstairs finding* = 0	
Barthell Index Scoring Add up the score: 20 = no disability 0 = complete disability	
Frailty Instrument: Katz – ADLs	
Count the number of: Each of the *able* qualified *ability to perform personal care activities finding* (bathing, dressing, toileting, feeding)(CCFOID:11244) = 1 *Unable* qualified = 0 Any *able* qualified *ability to transfer location finding* (CCFOID:1122) = 1 *Unable* qualified = 0 Both unqualified *continence finding* or *absent* qualified *incontinence finding* of either kind = 1 Unqualified *Incontinence finding* of either kind = 0	
Katz - ADLs Scoring Add up the score: 6 = high functioning 0 = low functioning	
Frailty Instrument: SF-36	
Average the following for General Health score: Questions 1, 33, 34, 35, 36 First assessment covers 5 questions, 1 score. unqualified or *high frequency* (CCFOID:441) qualified *fit and well finding* = 100 *mid-frequency* (CCFOID:442) qualified *fit and well finding* = 75 *absent* qualified *generally unwell finding* (CCFOID:1324) = 50 *mid-frequency* qualified *generally unwell finding* = 25 unqualified or *high frequency* qualified *generally unwell finding* = 0 Question 2 unqualified or *high frequency* qualified *fit and well finding* with *sudden onset* (CCFOID:433) qualification = 100 *mid-frequency* qualified *fit and well finding* with *sudden onset* qualification = 75 *absent* qualified *generally unwell finding* = 50 *generally unwell finding*: *mid-frequency* qualified = 25 unqualified or *high frequency* = 0 Average the following for Pain score Question 21 *absent* qualified *alteration in comfort: pain finding* (CCFOID:1111) = 100 *mid-frequency* qualified *alteration in comfort: pain finding* = 50 *high frequency* qualified *alteration in comfort: pain finding* = 0 Question 22 *absent* qualified *alteration in comfort: pain finding*, with *able* qualified *able to carry out daily routine finding* (CCFOID:11245) = 100 *mid-frequency* qualified *alteration in comfort: pain finding*, with *able* qualified *able to carry out daily routine finding* = 75 *high-frequency* qualified *alteration in comfort: pain finding,* with *able* qualified *able to carry out daily routine finding* = 50 *mid-frequency* qualified *alteration in comfort: pain finding*, *with difficulty* qualified *able to carry out daily routine finding* = 25 *high-frequency* qualified *alteration in comfort: pain finding*, with *unable* or *with difficulty* qualified *able to carry out daily routine finding* = 0 Average the following for Physical Functioning score: Question 3 *high frequency* qualified *enjoys vigorous exercise finding* (CCFOID:11233) or *able* qualified *ability to run finding* (CCFOID:11213) = 100 *mid-frequency* qualified *enjoys vigorous exercise finding* = 50 *gets no exercise finding* or *unable* qualified *ability to run finding* or *absent* qualified *enjoys vigorous exercise finding* = 0 Question 4 *high frequency* qualified *enjoys moderate exercise finding* (CCFOID:11232) = 100 *mid-frequency* qualified *enjoys moderate exercise finding* = 50 *gets no exercise finding* or *unable* qualified *ability to run finding* or *absent* qualified *enjoys moderate exercise finding* = 0 Question 5 *able* qualified *ability to perform general purpose physical activity finding* (CCFOID:11243) or *able* qualified *ability to perform shopping activities finding* (CCFOID:112413) = 100 *with difficulty* qualified *ability to perform general purpose physical activity finding* or *with difficulty* qualified *ability to perform shopping activities finding* = 50 *unable* qualified *ability to perform general purpose physical activity finding* or un*able* qualified *ability to perform shopping activities finding* = 0 Question 6 & 7 This covers 2 question (scores twice) *able* qualified *able to walk upstairs finding* = 200 *with difficulty* qualified *able to walk upstairs finding* = 100 *unable* qualified *able to walk upstairs finding* = 0 Question 8 *able* qualified *able to kneel finding* (CCFOID:11211) = 100 *with difficulty* qualified *able to kneel finding* = 50 *unable* qualified *able to kneel finding* = 0 Questions 9–11 This covers 3 question (scores three times) *able* qualified *able to walk finding* (CCFOID:112471) = 300 *with difficulty* qualified *able to walk finding* = 200 *unable* qualified *able to walk finding* = 0 Question 12 *able* qualified *ability to perform personal care activities finding* = 100 *with difficulty* qualified *ability to perform personal care activities finding* = 50 *unable* qualified *ability to perform personal care activities finding* = 0 Average the following for Role Limitations due to Physical Health score: Question 13–15 This covers 3 question (scores three times) *absent* qualified *occupational maladjustment finding* (CCFOID:1154) = 300 *mid-frequency* qualified *occupational maladjustment finding* = 150 *high-frequency* qualified *occupational maladjustment finding* = 0 Question 16 *able* qualified *able to carry out daily routine finding* = 100 *with difficulty* qualified *able to carry out daily routine finding* = 50 *unable* qualified *able to carry out daily routine finding* = 0 Average the following for Role Limitations due to Emotional Problems score Question 17–19 This covers 3 question (scores three times) *absent* qualified *occupational maladjustment finding* and any *psychological finding* (CCFOID:113) = 300 *mid-frequency* qualified *occupational maladjustment finding* and any *psychological finding* = 150 *high-frequency* qualified *occupational maladjustment finding* and any *psychological finding* = 0 Average the following for Energy/Fatigue score Questions 23, 27, 29, 31 (1 score) *able* qualified *able to sustain energy level finding* (CCFOID:132221) or *absent* qualified *lack of energy finding* or *absent* qualified *fatigue* = 100 *with difficulty* qualified *able to sustain energy level finding* or *mid-frequency* qualified *lack of energy finding* or *mid-frequency* qualified *fatigue* = 50 *unable* qualified *able to sustain energy level finding* or *high frequency* qualified *lack of energy finding* or *high frequency* qualified *fatigue* = 0 Average the following for Emotional Well-Being score Questions 24, 26 This covers 2 question (scores twice) *high frequency* qualified *calm finding* or *absent* qualified *nervous finding* or *absent* qualified *anxiety diagnosis* (CCFOID:21) = 200 *mid-frequency* qualified *calm finding* = 150 *mid-frequency* qualified *nervous finding* = 100 *absent* qualified *calm finding* = 50 *high frequency* qualified *nervous finding* or *anxiety* diagnosis = 0 Questions 25, 28, 30 This covers 3 question (scores three times) *high frequency* qualified *happy finding* or *absent* qualified *sad finding* (CCFOID:113334) or *absent* qualified *depression* diagnosis (CCFOID:22) = 300 *mid-frequency* qualified *happy finding* = 225 *mid-frequency* qualified *sad finding* = 150 *absent* qualified *happy finding* = 75 *high frequency* qualified *sad finding* or *depression* diagnosis = 0 Average the following for Social Functioning score Question 32 *able* qualified *ability to perform community living activities finding* (CCFOID:11241) = 100 *with difficulty* qualified *ability to perform community living activities finding* = 50 *unable* qualified *ability to perform community living activities finding* = 0 Question 20 *absent* qualified *impaired social interaction finding* (CCFOID:11531) = 100 *mid-frequency* qualified *impaired social interaction finding* = 50 *high frequency* qualified *impaired social interaction finding* = 0	
SF-36 Scoring Scores are from 0 to 100 for each section, higher score = less frail/better health	

In addition, we conducted preliminary NLP analysis using the ontology. We wanted to determine if narrative text that included frailty terms also included enough information to determine whether the patient had frailty-related functional deficits. Frailty terms from the Cardiac-centered Frailty Ontology and from a prior study [46] were used. We extracted 2460 clinical record snippets centered at the frailty keyword terms. Three clinicians and two informatics researchers reviewed the snippets. They categorized them as: a) Yes Deficit, or b) other. We trained a classifier on the snippets using a support vector machine (SVM). The average SVM performance, using 10-fold cross validation, achieved an accuracy score of 80.5%. Since frail patients typically have multiple frailty descriptions, the accuracy was deemed to adequately indicate that the terms in our ontology could be used to focus a learning system on frailty-relevant clinical text.

Finally, in [22] we tested whether the ontology could be used to help train a system to predict mortality for heart failure patients who underwent a major cardiovascular procedure (MCVP). We collected 2-years of clinical history data for a cohort of 20,000 heart failure patients leading to the MCVP. Frailty terms were identified in the text and classified as asserted or negated (i.e., “yes deficit” or “other”) using NLP. The ontology was used to map identified terms to their concepts. This study used an early draft of the ontology that had only 7 higher-level concepts: *therapy*, *medical findings*, *exercise*, *mobility*, *living activity*, *self-care* and *social function*. These concepts became *clinical findings*; *seen by professional allied to medicine*, *physical examination finding*, *activity exercise pattern*, *ability to move*, *activity of daily living*, *eating, feeding drinking ability*, and *social and personal history finding*, respectively. We aggregated the frailty concepts by group and selected maximum frailty score from among the concepts in each group.

**Fig. 2:**
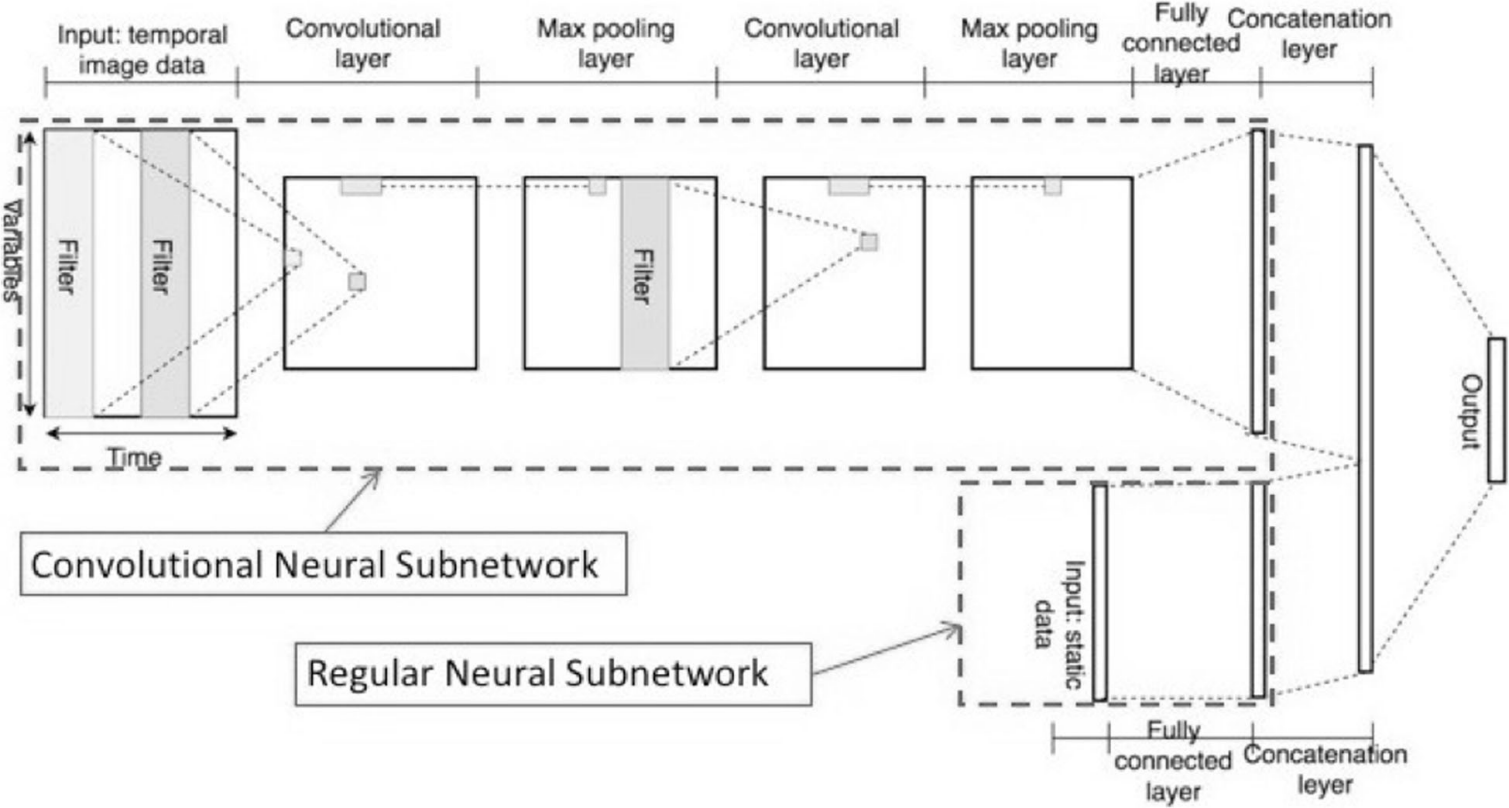
Deep neural network described in Zeng-Treitler, et al., 2018 [22]

A deep neural network (DNN), pictured in Fig. 2, was trained on a visual representation of the data features, which were hospitalizations, ICD9 codes for diagnoses, medications, and the frailty score. In ten-fold cross validation, the area under the curve (AUC) for mortality prediction was 78.3% (95% CI 77.1 to 79.5%) on the test data for the DNN model. We view this as additional validation for the ontology.

## Discussion

We developed and validated the Cardiac-centered Frailty Ontology. We created our own hierarchy to allow removal of unnecessary layers, unnecessary concepts, and maintain realist design principles as much as possible. We used SNOMED-CT concepts for all of the lowest level concepts. We incorporated 14 existing instruments in our initial development. We added five more for scoring and rule sets, when analysis of a 400-document clinical document set showed these instruments were in common use [20]. We adapted the standard ontology development model [32] by using clinician interviews to identify important concepts, without the necessity of forcing clinician agreement.

Our ontology development techniques differed from the standard techniques in two ways. We used vignette-guided interviews in lieu of a subject matter expert meeting to gain consensus and used validated frailty assessment instruments in lieu of the frailty literature. Interviews allowed us to determine concepts to assess patient frailty based on specific clinical decisions. That our participants came from different institutions helped minimized institution-related medical-cultural bias in frailty assessment. By including concepts that any one group might have excluded, we retain the chance that our NLP system will find all relevant concepts. Since the research group determined the concepts to include, we needed to be sure that we were not injecting our own opinions. Based on the amount of previous work in the area and existing SNOMED-CT concepts we included, we felt that our choices were not influenced by our opinions. We had our participants separately rate concepts for frailty severity, chance of cardiac intervention alleviating the problem, and relevance to frailty. By returning to the participants, we made explicit the extent and nature of their disagreements. We can then use this information going forward.

To address the concepts of frailty related cardiac interventions specifically; we included the concepts found in our interviews that we had not found from other sources. *Quadriceps weakness finding* is particularly relevant to cardiac intervention decisions because post-surgical patients cannot use their arms to help themselves stand. Surgical incisions require the upper body not be used. Therefore, if the patient cannot stand using their quadriceps alone, their post-surgical mobility is impaired, which impedes healing. Our participant ratings show that only a few concepts are specifically relevant to cardiac decisions, while around half are generally relevant, but not specifically relevant to cardiology. All four of the cardiac-specific concepts were also considered highly relevant to general assessments of frailty. Assessment of the utility of these cardiac-specific concepts in predicting patient outcomes was piloted as part of two studies to predict patient mortality [22, 47].

The Cardiac-centered Frailty Ontology reflects general frailty assessment as implemented in the frailty instruments used in its development [2, 48–58]. It includes participant ratings and our separate analysis of the interview data to create a picture of clinical decision-making with respect to cardiac interventions. Based on our orientation toward surgery-related decision-making [8–11, 13–17], we have excluded some of the specificity required to make non-surgery-related frailty decisions. We grouped all types of *lack of energy findings* together even though difference between “tiredness” and “weariness” may be important in other contexts. Once we have the NLP system functioning, it will be important to assess differences in outcome prediction using STS scores, with and without Cardiac-centered Frailty Ontology concepts.

We used a realist ontology development process [59] because it appeals to our understanding of the world, it helps ensure that the ontology is stable, and it avoids illogical inferences. By separating concept name and term list, we allow for language evolution, because the way *terms* are used changes over time. However, the portions of reality denoted by the concept and the concept name do not change.

Concepts refer to *Representations* in the minds of clinicians. These *representations* are far richer than the terms used to indicate their presence in the mind of an author. *Representations* are multi-modal. They include memories and imaginings, relevance to goals, and other information value attributes.

We are looking for clinical *findings*, which are conclusions drawn by clinicians and recorded in narrative form [60]. Restricting ourselves to *findings* also minimizes problems with illogical inferences. Take for example the concept *enjoys light exercise finding*, the truth of this as a conclusion drawn by a clinician is unchanged by whether or not the patient “enjoys” the process of exercising or whether or not the patient actually exercises. That it is an *activity exercise pattern finding* also remains a valid inference.

Our main focus was the findings noted in narrative text documented during clinical care, i.e., *clinical history findings*. We recognize that comorbid conditions are relevant to frailty assessment, but there are existent tools for identifying comorbid conditions. Clinical history findings represent the frailty-specific information we are interested in automatically extracting from clinical documents. We included a very restricted subset of *findings* from *physical examination*. We tested the comprehensiveness of our coverage by creating scoring rules for the frailty assessment instruments. If concepts were missing, we would not be able to create appropriate rules. We assessed the relevance of each concept by asking participants to rate them as high, medium, or low in relevance to assessing frailty with respect to cardiac intervention decisions. A preponderance of low relevance ratings would indicate a problem. We found only three. Three quarters of the concepts were rated as highly relevant. Taken together these results indicate that we have covered the necessary and sufficient concepts related to frailty assessment.

One of the best qualities of both SNOMED-CT [21] and the UMLS Metathesaurus [40] is their exhaustive coverage of the medical domain. One would be hard pressed to find a medical concept that was not contained within them. This exhaustiveness creates problems when we try to use them in NLP applications. Simple matching to either vocabulary results in too many false positives. The Cardiac-centered Frailty Ontology creates a comprehensive picture of frailty, while limiting the concepts from SNOMED-CT to only those directly relevant. We used concepts, with semantic type *finding*, found by human review of frailty assessment instruments, physician interviews, and chart review.

### Limitations

The main limitation of this work is the influence imparted on the ontology by our own ideas and biases. This limitation is shared by all ontologies. Our personal bias was minimized by the inclusion of the current accepted validated instruments on frailty. Each instrument reflects both expert consensus on the relevant concepts and empirical evidence of validity.

As is the case with all ontology development for NLP, ontologies precede NLP systems. The clinical outcome prediction NLP system used in our validation was not designed to model clinician decision-making. Without having a decision-making NLP system, it is difficult to assess whether the Cardiac-centered Frailty Ontology will facilitate all of the outcome predictions that we envision.

## Conclusions

We developed and validated a Cardiac-centered Frailty Ontology. The ontology is a machine-interoperable description of frailty that reflects all the areas that clinicians consider when deciding which cardiac intervention will best serve the patient. It was designed to share as many elements as possible with SNOMED-CT to allow interoperability. It could not be simply a subset of SNOMED-CT because there was no appropriate subset for us to choose.

## Methods

We used the ontology development process described in Noy, et al. [32]. This process consisted of four phases. *Phase 1* used existing ontologies and official clinical tools to identify individual terms. The clinical tools we used were validated frailty instruments and automated chart review. We expanded this to term based on physician interviews. In *Phase 2* we grouped terms into high-level concepts. We did this by examining concepts and hierarchies found in the existing SNOMED-CT ontology, while keeping the structure compact and realist. In *Phase 3* we defined object properties for concepts. Our methodology included mapping concept attributes from scoring collected for the identified concepts and properties indicated by instrument questions. Instruments have an associated property, which indicates a mapping between instrument scores and our ontology’s concept frailty scores. For *Phase 4*, analysis and validation, we created implementation rules for using the Cardiac-centered Frailty Ontology to reconcile scores on three common frailty instruments. Ontology structure was developed in Protégé [61], while term mappings were kept and shared in a Google sheet.

### Phase 1 – Aggregate terms form other ontologies and validated clinical tools

To extract frailty concepts from existing instruments, five members of the research team reviewed the specific items from 14 instruments chosen by the number of times they were cited and expert recommendation [20]: (1) Physical Frailty Phenotype (PFP, also called CHS frailty phenotype) [2]; (2) SF-36 [48]; (3) FIM [49]; (5) Clinical Frailty Scale [50]; (6) Brief Frailty Instrument [62]; (6) the Barthell Index [51]; (7) Health Assessment Questionnaire (HAQ) [52]; (8) PSMS [53]; (9) Katz ADL [54]; (10) Duke Activity Index [55]; (11) RDRS [56]; (12) FACIT [57]; (13) NYHA [58]; (14) Deficit Accumulation Index (DAI, also called Frailty Index) [63]. Each person reviewed each individual item from each instrument. Terms from the World Health Organization’s International Classification of Functioning, Disability and Health (ICF) were also included in the analysis because the instruments varied in their levels of abstraction, their scopes, and uniqueness.

At this step we added an ontology entry for comorbid condition count. Comorbid conditions are an important indicator of frailty. However, they are not the focus of our investigations. We focused on frailty-specific indicators in order to identify core frailty concepts in clinical documents.

The concept list was expanded by the findings of the interviews of cardiologist and cardiac surgeons described above.

Finally, we included terms extracted from manual note review by members of the research team with clinical experience. These reviews were in preparation for NLP topic modeling by Shao, et al., (2016). They reviewed clinical notes and social media posts [64].

### Phase 2 - group terms into high-level concepts

We organized constructs in hierarchical relationship based on: 1) the results of topic modeling, 2) the basic organization of the frailty instruments, and 3) by mapping them to SNOMED-CT equivalents. We used SNOMED-CT concepts within the category *clinical finding*, with semantic type *finding*. The goal for this process was to restrict the SNOMED-CT mappings to as small a selection as possible, while maintaining correspondence with groupings from topic modeling and instruments, which means our ontology is somewhat compatible with SNOMED-CT.

### Phase 3 – Define object properties for concepts

Object properties were determined in two ways, through the scales used to answer instrument questions and by scoring terms and concepts based on key decisions when making cardiac surgery decisions. For *instrument findings*, we defined properties, which related instrument scores to the Rockwood global assessment of frailty (described below) [45].

Rockwood categories are described in the Dalhousie University Clinical Frailty score, which has 9 categories [45]. The categories are (1) very fit, (2) well, (3) managing well, (4) vulnerable, (5) mildly frail, (6) moderately frail, (7) severely frail, (8) very severely frail, and (9) terminally ill [50]. Concepts in the ontology vary in how they map onto these severity categories. Some concepts have three levels of severity of impairment (able, assisted, and unable). The concept *ability to walk,* for example, has these three levels, where each level indicates a different Rockwood category. The scales provided for question answering in the frailty instruments indicated concept severity levels. These scales took the form of *able* to *unable* and *all the time* to *never*.

To establish the relationship of the concepts to these aspects of frailty three members of the team (BB, CW, KDH) and 3 participant cardiologists scored the concepts on three key decisions identified in the interviews: 1) Rockwood category (described below) as an indicator of ability to survive surgery, 2) relevance to cardiac decision-making as a reflection of the patient’s ability to recover from surgery, and 3) the likelihood that the cardiac intervention will fix the problem.

Another outcome of our physician interviews was that clinicians consider indications of frailty within the context of cardiac decisions by assessing whether they are likely to be a result of the patient’s cardiac condition and whether they are specifically relevant to cardiac decisions. The medically trained members of this group also characterized the constructs as low, medium, high for both their likelihood to be fixed by cardiac intervention and their relationship to cardiac decision-making.

For instrument scores, we created score rating for 10 commonly cited instruments that were not included in the initial concept-finding step. The initial mapping was created by author YC and verified by the remaining authors.

### Phase 4 – Analyze and validate

For this phase, we created rules to implement three frailty assessment instruments using the Cardiac-centered Frailty Ontology. We mapped instrument questions and responses to Cardiac-centered Frailty Ontology concepts and properties.

We also tested the utility of the ontology in two different automated NLP systems. One system was designed to classify clinical note snippets as indicative or frailty or not. The other was designed to predict patient mortality after MCVP.

## Additional file

Additional file 1: Frailty ontology concept list. (XLSX 37 kb)

## Abbreviations

ADLs: Activities of daily living
BMI: Body mass index
CABG: Coronary artery bypass graft
CCFOID: Cardiac-centered frailty ontology identification number
DNN: Deep neural network
EHR: Electronic Health Record
FAQ: Functional activity questionnaire
FIM: Functional independence measure
IADL: Instrumental activities of daily living
MCVP: Major cardiovascular procedure
MRT: Morse fall scale
NLP: Natural Language Processing
OGMS: Ontology of General Medical Science
SCTID: SNOMED-CT identification number
SF-36: MOS 36-item short form health survey
STS: Society of Thoracic Surgeons
SVM: Support vector machine
TAVR: Transcatheter valve replacement
UMLS: Unified Medical Language System
VA: Veterans affairs
